# A Review on Initiatives for Promoting Better Menstrual Hygiene Practices and Management in India

**DOI:** 10.7759/cureus.47156

**Published:** 2023-10-16

**Authors:** Shambhavi Kumari, Komal N Muneshwar

**Affiliations:** 1 Department of Community Medicine, Jawaharlal Nehru Medical College, Datta Meghe Institute of Higher Education and Research, Wardha, IND

**Keywords:** waste, hazards, sanitation, infections, hygiene, menstruation

## Abstract

Management of hygiene during menstruation is severely affected by the continued existence of several societal, cultural, and religious restrictions on menstruation and menstruation practices. Since they are frequently unprepared and unaware of their periods, especially in rural regions of the country, girls face a variety of hurdles and problems at home, in school, and at work. We observed from examining the studies that managing menstrual hygiene is affected by a lack of personal sanitation and inadequate, erroneous, or partial understanding of menstruation. Women either possess zero or minimal knowledge about genital infections, which are caused by poor personal hygiene during menstruation. Women in rural areas frequently lack access to sanitary items and have inadequate understanding of their sorts and applications, or cannot afford the high cost of such products. Females in rural areas must use washable cotton pads because of this, which they must use repeatedly. The requirements and desires of teenage girls and women continue to be disregarded, despite substantial developments in the water and sanitation industries. Menstrual products are disposed of in domestic garbage and public facilities when women are outside, while they are flushed down toilets at home without considering the risk of choking. Because of this, there should be a need to inform and educate individuals about the health risks and environmental problems connected to them. Reduced trash may be achieved by using modern techniques like incineration. Therefore, the importance of using natural or reusable sanitary products should be emphasized.

## Introduction and background

Young female and premenopausal women physiologically experience menstruation. From puberty till menopause, it is characterized by the regular flow of blood through the vagina from the uterus, which is caused by the uterine lining shedding. The average menstrual cycle lasts 21 to 35 days and is brought on by hormones [1]. The two distinct phases of the menstrual cycle that may be distinguished are the luteal or secretory phase and the follicular or proliferative phase. The number of days between the start of menses on one cycle and the first day of menstruation on the following cycle is the duration of a menstrual cycle [2]. The most significant element affecting a woman's reproductive health is her menstrual cycle. Menarche is a prominent indication of puberty and a significant event in adolescent girls' lives. Menstrual issues are a typical symptom and one of the main reasons why adolescents visit the doctor. Hence, girls understanding of reproductive health, including menstruation, may be lacking, and influenced by sociocultural barriers. In India, most teenage girls have no idea about menstruation, reproduction, or sexuality. Menstruation is still considered taboo; thus, the culture upholds many misconceptions and restrictions.

Reproductive tract infections (RTIs) and gynaecological problems are more likely to occur during menstruation due to poor personal hygiene and unsafe sanitary conditions [3]. It is typical for cycles to be erratic and protracted during the first several years after menarche. Polymenorrhagia, oligomenorrhea, and dysmenorrhea are the three most prevalent menstrual disorders. In the first few years of menstruation, irregular periods are more common; however, they are less common after the first three to five years following menarche. Due to the possibility that women may increase their susceptibility to RTIs, women's hygiene-related behaviours during menstruation are crucial. Poor menstrual hygiene is a significant factor in female morbidity and one of the key causes of the high prevalence of RTIs in the country [4]. Menstruation and related terms, including myths and taboos, are overlooked due to a lack of awareness. Many girls in India between the ages of 10 and 18 drop out of school due to a lack of access to menstrual hygiene facilities [5]. Poor economic status, scarcity of resources, and the perception of menstruating females as dirty all play a role in this predicament. Many girls have been denied access to their fundamental right to education because of the stigma surrounding menstruation difficulties. This leads to the rise in illiteracy, prejudice, and menstrual myths, among other things. Most issues with menstruation health and access to services are experienced by women in rural and underdeveloped sections of the nation. Consequently, there is a need to raise awareness among people about menstruation [5].

## Review

Methodology

**Figure 1:**
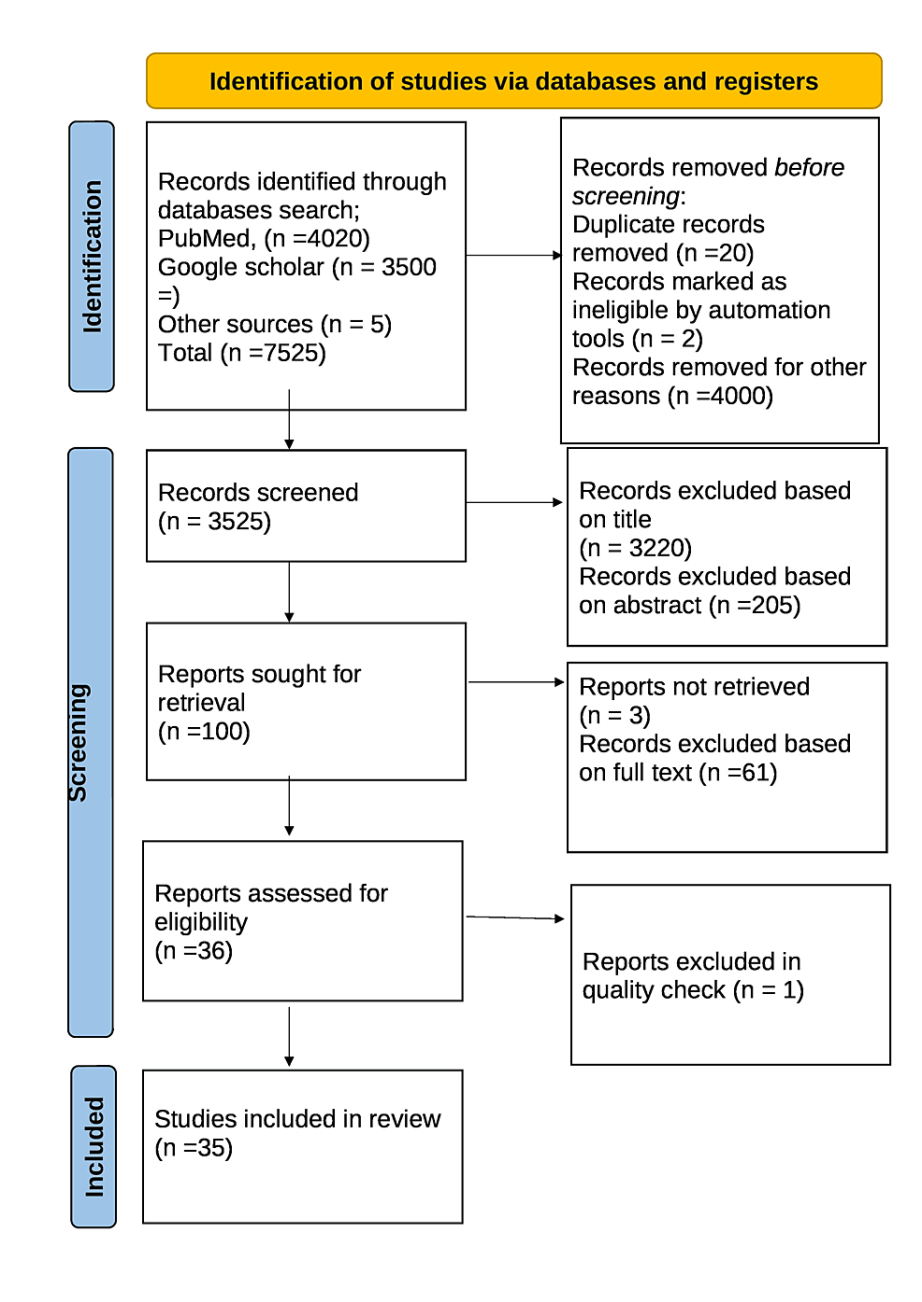
PRISMA flow diagram Adapted from PRISMA flow diagram 2020. PRISMA: Preferred Reporting Items for Systematic Reviews and Meta-Analyses

A search was conducted in databases PubMed, Google Scholar, and Scopus using key terms “Menstrual hygiene practices in India” or “initiatives for better menstrual hygiene practices” or “menstrual hygiene management” were the search phrases. Related articles over a period of the last 10 years were searched which included free full text, reviews, book articles, website reports and online published reports. About 65 articles were obtained. After screening for duplicates, suitability, inclusion, and exclusion criteria, a total of 65 articles were shortlisted. A total of 35 articles were included in the final review. Figure 1 shows the Preferred Reporting Items for Systematic Reviews and Meta-Analyses (PRISMA) flow diagram for searched articles.

Unhygienic strategy adopted during menstruation

There have been reports of unsanitary behaviours during menstruation, such as changing bathing routines and using unclean materials to absorb blood [6]. Women in isolated areas sometimes need access to sanitary products, know little about the types and uses of such products, or cannot afford the high cost of those products. As a result, they primarily rely on washable, reusable pads [7]. Traditionally, menstrual flow has been absorbed by clothes since they are less costly and environmentally damaging. Nevertheless, particularly in urban areas, pads are progressively replacing cloths. Women may find cleaning and storing their clothes challenging if they need access to water, privacy, and a drying area [8]. However, since washing occurs without soap and with unclean water, as well as because indoor drying is required away from sunlight and open air due to social taboos, reusable materials cannot be adequately sanitized. Unhygienic washing habits are particularly prevalent among women and girls in lower socioeconomic strata and rural regions. Menstrual flow since they are less costly and less harmful to the environment. However, pads are gradually taking their place, especially in metropolitan areas. If women need access to water, privacy, or a drying room, cleaning and drying clothes may be challenging [9]. Menstrual hygiene management is likely to be impacted by external factors, such as the accessibility of areas where women may clean up after their periods in privacy and comfort. The availability of water, hygiene, and sanitation facilities within the place impacts these variables. RTIs might be more common in women with poorly managed menstrual hygiene. It has been seen that women who use unsanitary menstrual hygiene management practices are more likely to get bacterial vaginosis [9].

Menstruation restrictions and cultural beliefs

Culture-specific customs, influence from families, individual preferences, financial conditions, and pressure from society all had an impact on how people handled their periods. The misconceptions and attitudes about menstruation prevalent in a particular culture or religion are menstrual beliefs. Menstrual hygiene management was tied to menstrual beliefs, knowledge, and practices [8]. These norms hindered the proper menstrual hygiene practices. Many women are prohibited from doing certain things, including cooking, working, having sexual relations, bathing, worshipping, and eating certain foods. These limits were imposed owing to the general public's negative opinion of menstruation, which they believe is unclean and polluting [9]. Due to having their period, many young women and girls have limits in their daily lives. The primary restriction for women in urban areas during menstruation is to avoid the "puja" room, but for girls in rural areas, it is to avoid the kitchen. Menstruating women and girls are also prohibited from praying or touching holy items. The underlying assumption of this myth is similarly established in cultural perceptions about the impurity of menstruation.

Furthermore, it's thought that because menstruating women are impure and unclean, the food they handle or prepare might get tainted [10]. Menstrual cycle restrictions influence women's and girls' attitudes, way of life, and, most importantly, health. These limits are typical in many cultures. When their period begins, a sizable fraction of female students drop out of school in many less developed countries. It involves approximately one-fourth of Indian girls. The monthly menstrual cycle also poses difficulties for female teachers. The absence of suitable menstruation protection options, clean, safe, and private sanitary facilities for female educators and learners, and the culture and setting of gender-exclusive schools compromise the right to privacy. There are additional health and hygienic considerations concerning girls and menstruation [11].

**Figure 2:**
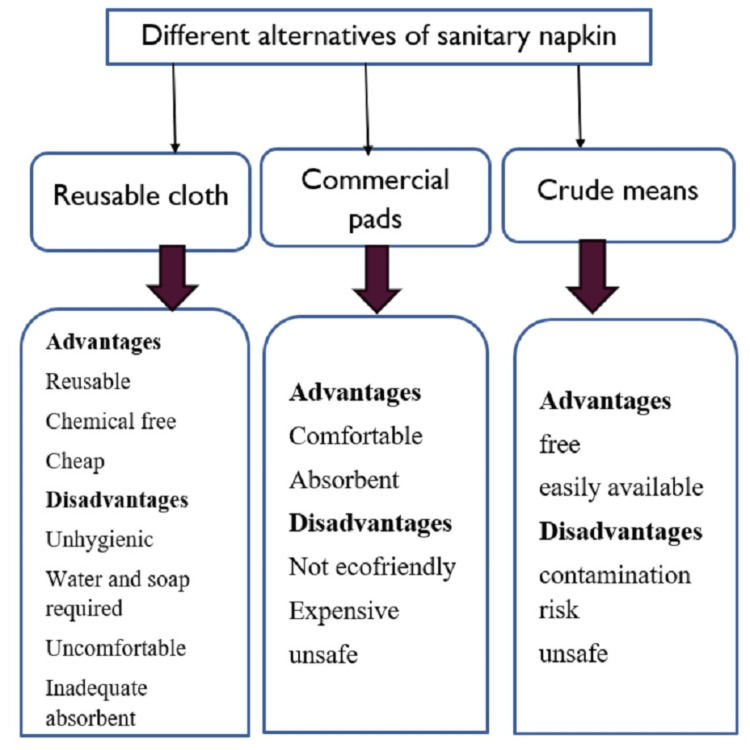
Types of materials used during menstruation Self-created image

Figure 2 shows the types of materials used during menstruation.

Types of materials used during menstruation

Personal preference, cultural tolerance, economic standing, and market availability all play a role in determining the preferred sanitary protection material. Menstrual absorbents and soap should also be available, in addition to the essential sanitation facilities, to help manage menstruation hygiene. Urban and rural women and girls prefer different absorbents. On the market, there are two categories of menstruation products: (a) disposable, or those that are only used once, like tampons and disposable pads; and (b) reusable items, like cloth, menstrual cups and pads that can be washed and reused [12]. Sanitary pads are conveniently available at various retail locations, pharmacies, and online. They are more costly than cotton pads, one-time use only, and not very eco-friendly. They were made using cotton that was not entirely natural and could have pesticides. Because sanitary napkins can take several years to decompose, using them poses a serious environmental risk. In addition, 2 g of plastic is left behind from each sanitary napkin that cannot biodegrade. Monthly massive amounts of plastic are going to accumulate as more women throughout the world menstruate. Dioxin, which can cause pollution and other adverse environmental effects, can be produced when the pads are burned. Infections can be developed in longer-used pads that are left exposed or in touch with the air. The accumulation of moisture might lead to a fungus infection [13].

Tampon

They are within the category of absorbents that offer protection. To absorb the menstrual flow before it exits the body, they are a soft cotton plug put into the vagina. They are expensive, not naturally biodegradable, and therefore not environmentally friendly. Toxic shock syndrome, which can result in death from the bacteria *Staphylococcus aureus*, often known as staph, invading the body, can be brought on by tampons left in the vaginal area for an extended time [14].

Cloth Pad

Cloth pads are an environmentally friendly sanitary alternative but must be adequately cleaned and dried outside in the sun. The sun's warmth is a natural sterilizer; drying cloths and pads beneath it sterilize them for upcoming usage. Because these are reusable, they are affordable, widely accessible, and environmentally beneficial. Additionally, they must be kept clean and dry while not in use to prevent infection [15].

Initiatives adopted for better menstrual hygiene practices

Some teenagers wait until their menstrual cycle is finished at home due to the growing costs of sanitary pads. This shows that people in many parts of India cannot afford clean items, preventing them from properly managing their menstrual hygiene [16]. A pack of monthly pads is equivalent to four plastic bags due to a pad's estimated 90% plastic content. Plastic is also present in tampons, string, and plastic applicators of polyethene and polypropylene. The feminine hygiene product leaves behind a significant carbon imprint. Due to the non-biodegradable nature of the plastic disposed of in a landfill and releases gases dangerous to human health and the environment, one sanitary pad can endure 500 and 800 years, according to the Menstrual Health Alliance India. Most businesses concentrate on producing sanitary pads but often neglect to consider how their disposal would affect the planet [17]. Therefore, it is imperative to switch from expensive, non-biodegradable sanitary products to affordable, biodegradable alternatives that are accessible to all women in India's rural and urban areas. Various programmes are underway to improve menstruation hygiene, and one of them will look at how frequently available materials made of biodegradable natural fibres may be used as sanitary pads. Managing menstruation hygiene might be made more accessible for billions of women in low- and middle-income nations [18].

Plant-Based Sanitary Pad

The long, smooth, and lustrous vegetable fibre known as jute may be woven into thick, durable threads. It is one of the most economical natural fibres in manufacturing and applications, just next to cotton. The plant substances cellulose and lignin make up most of the fibres in jute. It is also known as the "golden fibre" because of its golden tint and significant economic worth. It is possible to substitute petroleum-based raw materials with ecologically friendly ones by using jute fibre, which has a low carbon imprint and produces sanitary goods that are highly sustainable. An eco-friendly world will result from the widespread usage of jute fibre in hygiene products [19].

Another extremely absorbent substance is bamboo fibre or bamboo cloth. Additionally, bamboo fibre soaks more than cotton. A bamboo cotton combination has a higher absorption capacity than pure cotton, while pure bamboo has the highest antibacterial action. The bamboo fibber's cross-section is dotted with a lot of tiny gaps and holes. Unlike other natural materials, the cellulose in these fibres has crystalline and hierarchical structures [20]. A special bio-agent known as "Bamboo Kun" that inhibits bacteria growth and is antibacterial has also been discovered in bamboo. This fibre makes sense for sanitary items because it will not harbour as much bacteria as other options when worn for lengthy periods. It is very absorbent, recyclable, has good airflow, and has several antimicrobial qualities, so it is a tremendous sanitary replacement. Bamboo wadding is the most suited material for clean items since it is approximately twice as absorbent as a commercial sanitary pad, incredibly absorbent, inexpensive, lightweight, and biodegradable. It has no negative impacts on the user or the environment [21].

A floating aquatic plant known as a water hyacinth is invasive in many areas. This plant reproduces asexually, producing daughter plants with linked root systems by stolons. It develops exceptionally swiftly, with a doubling time of around two weeks. As a result, water hyacinths can thrive in an environment that is hindered by floating barriers in the water. When growing in large numbers, water hyacinths can harm aquatic life by lowering water oxygen levels and displacing native plant species. The water hyacinth not only obstructs marine life but also restricts, if not completely prevents, recreational usage of the body of water. Fibres from water hyacinths have been used to make sanitary products. It is a good substitute for plastic-based pads since it is made of cellulose, degrades naturally, and is safe for use on people [22].

Bananas and bamboo fibres are supplied locally by Saathi, the company that made the Saathi Pad. Although this pad was made in Gujarat, India, it may be purchased by menstruating women elsewhere. These pads' only ingredients are plant-based, making them chemical-free and biodegradable. This reduces the risks they represent to the environment and human health compared to pads made of plastic. The pad expands the supply of pads for women in India and fosters a culture of menstruation knowledge among customers [23]. It also hires women to promote gender equality, transforming this region's economic and social environment and culture. It also boosts the local economy and increases access to inexpensive sanitary pads by buying supplies from nearby farmers and selling pads at a very reasonable rate. Finally, safe developed a reusable, biodegradable pad to solve the social and environmental problems associated with menstruation and basic menstrual hygiene. The reusable pad aids menstruating women in India in using good hygienic practices. It was made from banana fibre, cotton polyurethane laminate, and polyester piling. Banana fibres are used in the creation of these pads to cut production and market costs, making the pads more affordable for women [24].

Other initiatives

Menstrual Cup

India sells menstrual or vaginal cups. It does not cause infections, allergies, or rashes because it comprises non-absorbent silicone rubber. Because it is worn inside, it does not produce any unpleasant odours or a damp feeling. Before use, it should be sterilized. The menstrual cup can help low-income people who can't afford premium sanitary products because of its minimal risk and comfortability [25]. These are more cost-effective, reusable, and durable than sanitary pads. Compared to a single cup, which costs around 300 rupees and can be used for more than five years, they had to spend about 50 rupees monthly on sanitary pads to last for just one cycle. Due to persisting taboos around menstruation and virginity, young unmarried ladies might not feel safe inserting the cup. Furthermore, utilizing it would be difficult without toilets with enough water supply. Additionally, these are not often used because of a lack of understanding among women in the reproductive age range, a lack of awareness campaigns, and a lack of commercial ads [26].

Coimbatore-based social entrepreneur Arunachalam Muruganantham created an inexpensive sanitary pad manufacturing equipment. Through his devices, he revolutionized menstruation hygiene for women in rural India. He effectively distributes affordable sanitary pads to 4,000 Indian communities [27]. His uncomplicated inventions and the method by which any woman in the world may make them allow sanitary pads to be produced for less than a third of the price of commercial pads. In only one and a half years, he manufactured around 240 machines. He delivered them to some of Northern India's poorest and least developed regions, such as Bihar, Madhya Pradesh, Rajasthan, and Uttar Pradesh, where women must travel great distances to get water when menstruating, putting their families through hardship [28].

Disposal of sanitary napkins

In most places in India, there needs to be more proper toilets and sanitation facilities to dispose of sanitary products, making it difficult for women to manage menstrual hygiene. The most popular unsafe practice for disposing of menstruation-absorbent materials is dumping them unwrapped on roofs in fields. Place outside after wrapping in paper or a plastic bag. Drying, wrapping in paper or a plastic bag, putting in trash cans, and burying for decomposition. Due to the lack of proper disposable facilities, women often throw dirty pads wrapped or unwrapped in the public restroom or bins directly, even after knowing the harmful effects of doing this. This renders the bathrooms unsanitary for frequent users and maintenance staff, a shelter for insects and flies, and a breeding ground for disease. Due to insufficient water supply, damaged toilet locks/doors, absence of water taps, and lack of disposal systems, several girls were claimed to have missed school [29].

Techniques to improve management

In India, a significant amount of stigma is attached to menstruation, making it challenging to handle sanitary waste properly. If managed improperly, this infectious waste would constitute a severe hazard to the health of people, the environment, and the seas. While encouraging the use of sanitary napkins has received most of the attention to date, it is necessary to address the issues brought on by disposing of pure waste in landfills. It is essential to develop a sustainable solution regarding the multiple risks brought on by this extensively utilized but problematic disposal approach. A decentralized method of handling menstrual waste at the place of creation is essential to prevent the problem from worsening. Promoting women's health and dignity is crucial to designing restrooms that support menstrual hygiene practises, providing availability of absorbents, and encouraging safe handling and disposal of used absorbents [30]. Menstrual wastes should be collected separately without compromising women's privacy or sense of worth. It is advisable to install specific sanitary dispensers for collecting menstrual waste. Enough room should be provided for washing, cleansing hands and intimate areas, changing clothes, and handling soiled clothing. To meet these criteria, there must be access to water, toilet paper, a trash can, and a sink where menstruation items may be washed. To prevent flies, mosquitoes, and foul odours from entering the restrooms, the dustbins should be covered with a lid and periodically emptied [31]. The Indian government is promoting menstrual waste burning as a means of disposal. If incinerators adhere to design and emission guidelines, this approach might assist in lessening the environmental impact of menstrual waste. Menstrual waste management options include burning, particularly in the workplace, educational, and dormitory environments. When deciding which type of incinerator to use, several factors need to be taken into account, such as the absorbents that will be used and their makeup, the location of the incinerator (such as a home, public restroom, or an institution), the amount of waste that needs to be burned, the temperatures at which the appliance will be treated, its capacity, and its emissions, as well as the budget that can be allocated and its ability to be operated and maintained. The majority of time should be spent burning non-biodegradable sanitary pads [32].

Discussion

Table 1 shows the summary of the characteristics of the included studies.

**Table 1:** Summary of the characteristics of the included studies Self-created table

	Article type	Country/trial	Findings
Kpodo L et al. [1]	Review	Ghana	A detailed definition of menstruation.
Mihim M et al. [2]	Review	India	A detailed physiological aspect related to menstruation was done.
Dar MA et al. [3]	Review	India	The study shed light regarding menstrual issues.
Singh AJ et al. [4]	Review	North India	Importance of maintaining hygiene during menstruation.
Ramteke R et al. [5]	Review	India	The study reflected on various myths and stigmas related to menstruation.
Ali TS et al. [6]	Review	India	The study reflected on how unhygienic practices are adopted by women.
Kaur R et al. [7]	Review	India	Studies showed issues related to menstruation in rural areas.
Kumar K et al. [8]	Review	India	Studies showed how sanitary pad is more valuable than clothing pad.
Garg S et al. [9]	Review	India	Study reflected on proper management during menstruation.
Thakuri DS et al. [10]	Review	Nepal	Study showed restrictions faced by women during menstruation in different areas.
Roberts TA et al. [11]	Review	USA	About difficulties faced by school-going girls and working women.
Ejik AM van et al. [12]	Review	India	Different categories of menstrual products.
Patel K et al. [13]	Review	India	Study showed hazardous effects of plastic sanitary napkins.
Billon A et al. [14]	Review	France	The drawbacks and benefits of tampon was shown.
D'allesandro D et al. [15]	Review	USA	Studies show about proper use of cloth pads.
Asumah MN et al. [16]	Review	Ghana	Mismanagement of menstrual hygiene products.
Ghosh I et al. [17]	Review	India	Study shows how sanitary pads are composed of plastic, which are harmful for the environment.
Sivakami M et al. [18]	Review	India	About switching to affordable biodegradable from expensive non-biodegradable products.
Agbaku CA et al. [19]	Review	China	About jute as an option for sanitary pad production.
Foster J et al. [20]	Review	USA	About bamboo fibre as a core of sanitary products.
Barman A et al. [21]	Review	India	Reflection on benefits of bamboo as a core of sanitary products.
Kipchumba BB, Kulei AK, Mwasiagi JI [22]	Review	India	About water hyacinth as a substitute for sanitary products.
Tudu PN et al. [23]	Review	India	About banana and bamboo as an option to use.
Peter A et al. [24]	Review	India	About menstrual cups and its benefits.
Kaur H et al. [25]	Review	India	Showed various benefits of using banana fibre in making menstrual products.
Somani P [26]	Review	India	About organisation putting light on menstrual hygiene.
Van Ejik AM et al. [27]	Review	UK	Studies have shown how menstrual cups can be harmful for women.
Chatterjee P et al. [28]	Review	India	About invention of inexpensive pad by Arunanchalam Muruganantham and his contribution on menstrual products.
Elledge MF et al. [29]	Review	India	Reflection on ill management of menstrual waste disposal and hygiene management in low and middle-income countries.
Bhor G et al. [30]	Review	India	Adverse affect of improper management of menstrual waste disposal.
Kumar B et al. [31]	Review	India	It shows the proper technique of menstrual waste products disposal and harmful substance found in sanitary napkins.
Roy A et al. [32]	Review	India	About necessity for education regarding menstruation.
Banappagoudar SB et al. [33]	Review	India	It tells about proper use of menstrual napkins for maintaining menstrual hygiene.
Choudhary N et al. [34]	Review	India	About hygiene practices during menstruation.
Sharma S et al. [35]	Review	India	Reflection on awareness and programmes on menstrual hygiene problems.

Result

Although menstruation is a natural phenomenon, society has not yet come to terms with it. There are several sociocultural perspectives on menstruation. Sometimes, people think of it as a disease or a heavenly curse. Menarche is revered, while monthly periods are taboo and seldom spoken publicly. All these things have negatively and perplexingly impacted young girls' attitudes regarding menstruation. This highlights the necessity for adequate education to modify views around menstruation [32]. Infections of the reproductive tract and gynaecological problems are more likely to occur during menstruation due to inadequate hygiene and unsafe sanitary conditions. The typical amount of time a woman should wear a sanitary napkin is 3 to 4 hours, after which the napkin must be replaced. Wearing a sanitary napkin for longer than 4 or 5 hours might result in rashes, irritation, itching, and swelling.

Heat and moisture in the vaginal region can occasionally promote bacterial growth, leading to infection and gynaecological problems. When the exterior skin of the vagina is irritated by a layer of the pad or by the substance used in the pads, such as the back sheet, fragrance, adhesives, etc., it results in contact dermatitis, which causes a rash [33]. Maintaining a good standard of cleanliness in areas with few resources is very challenging when women lack access to basic amenities like water, restrooms, and solitude. In terms of essential amenities, housing circumstances need to be improved. The use of sanitary pads should be promoted to all females. Girls may find it easier to cope and seek the proper medical care with the help of school-based sensitization, counselling, and comprehensive programmes on menarche and menstrual problems [34,35].

## Conclusions

The kind of product used, how often it is replaced, frequent washing, cleaning of the intimate area, as well as proper disposal of used products all affect menstrual hygiene. Use of clean materials, changing pads a minimum of two to three times daily, taking a daily bath, and washing the intimate area with water are examples of appropriate hygiene practices. Products used during menstruation have improved with time. Menstrual cups, tampons, and sanitary napkins are some of these consumables. The menstruation product used the most frequently is the sanitary napkin. There are now several eco-friendly, reusable napkins available. Even though only women menstruate, it is imperative that every member of society has a basic understanding of this. Menstrual hygiene is a crucial issue that has just lately started to get the attention it needs and is now being seen from several angles, including equality between men and women, sanitation, hygiene, and water supply, reproductive wellness, educational opportunities, and public health. This is a result of how it affects the condition of the environment, the finances, the overall labour market, and the growth of nations. Additionally, it affects the female child's safety as well as family, health, and education. Girls' required knowledge about physiological aspects of menstruation, the connection between menstruation diseases, symptoms, and fluctuations in hormones, as well as the contributing elements, will be raised through workshops and the introduction of a chapter to course materials that focuses on enhancing lifestyle and related variable elements. Considering these circumstances, it would be advisable to promote the manufacturing of completely natural and entirely safe sanitary napkins. We should promote the use of banana, cotton, hyacinth, bamboo, and oil cloth in sanitary napkins so that they are fully organic, won't irritate skin or produce a rash or redness, and are also soft and biodegradable in addition to being anti-microbial and antifungal.
